# Supplementary material for: Mechanisms governing the pioneering and redistribution capabilities of the non-classical pioneer PU.1
Source: Nat Commun. 2020 Jan 21;11:402. doi: 10.1038/s41467-019-13960-2 (PMC6972792; doi:10.1038/s41467-019-13960-2)
Supplement: Supplementary file 7 — Source data [file 41467_2019_13960_MOESM7_ESM.zip › Source_Data/FigureS2/FigureS2B_MotifScanOutput/KmeansCluster6/homerResults/motif1.similar.html]

motif1

## Information for motif1

A
G
T
C
C
T
G
A
A
T
G
C
C
G
A
T
A
C
G
T
A
G
T
C
A
G
T
C
A
G
C
T
A
G
T
C
G
C
A
T
G
C
A
T
G
A
C
T
  
Reverse Opposite:  

C
T
G
A
C
G
T
A
C
G
T
A
T
C
A
G
T
C
G
A
C
T
A
G
A
C
T
G
C
G
T
A
C
G
T
A
T
A
C
G
G
A
C
T
T
C
A
G
  

|  |  |
| --- | --- |
| p-value: | 1e-1720 |
| log p-value: | -3.961e+03 |
| Information Content per bp: | 1.636 |
| Number of Target Sequences with motif | 2882.0 |
| Percentage of Target Sequences with motif | 87.52% |
| Number of Background Sequences with motif | 6161.4 |
| Percentage of Background Sequences with motif | 14.32% |
| Average Position of motif in Targets | 100.9 +/- 36.0bp |
| Average Position of motif in Background | 101.1 +/- 84.0bp |
| Strand Bias (log2 ratio + to - strand density) | -0.0 |
| Multiplicity (# of sites on avg that occur together) | 1.59 |
| Motif File: | file (matrix) reverse opposite |

### Similar de novo motifs found

|  |  |  |  |  |  |  |  |
| --- | --- | --- | --- | --- | --- | --- | --- |
| Rank | Match Score | Redundant Motif | P-value | log P-value | % of Targets | % of Background | Motif file |
| 1 | 0.983 | A T G C C G T A A T G C C G A T A C G T A G T C A G T C G A C T A G T C G C A T G A C T | 1e-1631 | -3755.724100 | 88.92% | 16.70% | motif file (matrix) |
| 2 | 0.975 | C T G A C G T A T C A G T C G A T C A G A C T G C G T A G C T A T A C G G A C T | 1e-1559 | -3590.756610 | 85.55% | 15.36% | motif file (matrix) |
| 3 | 0.954 | C G T A C G T A C G T A T C A G C T G A A C T G A C T G C G T A C G T A T A C G G A C T C T G A T C G A | 1e-1531 | -3526.921613 | 86.21% | 16.28% | motif file (matrix) |
| 4 | 0.939 | A C G T A C G T A C T G C T G A A T G C A C G T A C G T A G T C A G T C A G C T A G T C G C T A G C T A G C A T | 1e-1423 | -3276.919143 | 77.98% | 12.57% | motif file (matrix) |
| 5 | 0.980 | C T G A C T G A C T G A T C A G C T G A C T A G A C T G C G T A C T G A T A C G G C A T T C A G T C A G | 1e-1393 | -3208.766435 | 69.36% | 8.49% | motif file (matrix) |
| 6 | 0.941 | C T G A A T G C C G A T C G A T A G T C A G T C A G T C G A T C G C A T G C A T G C A T | 1e-1363 | -3139.622176 | 93.81% | 26.51% | motif file (matrix) |
| 7 | 0.940 | C T G A A T G C C G A T G A C T G T A C A G T C G A C T A G T C G C A T G C A T G C T A G A C T | 1e-1319 | -3038.490204 | 93.47% | 27.15% | motif file (matrix) |
| 8 | 0.916 | C G T A A T G C A C G T A C G T A G T C A G T C A G C T A G T C | 1e-1273 | -2932.961206 | 86.00% | 20.84% | motif file (matrix) |
| 9 | 0.936 | C T A G T C G A C T A G A C T G C G T A C G T A T A C G G C A T T C A G | 1e-1246 | -2870.672217 | 77.01% | 14.73% | motif file (matrix) |
| 10 | 0.963 | A G T C G A T C G C T A A T G C G A C T G A C T A G T C G A T C G A C T A G T C G A C T G A C T A G C T G A C T | 1e-1217 | -2804.309490 | 70.24% | 11.22% | motif file (matrix) |
| 11 | 0.932 | C G T A C G T A T C A G T C G A C T A G A C T G C G T A G T C A T A G C | 1e-1207 | -2781.249187 | 84.51% | 20.96% | motif file (matrix) |
| 12 | 0.893 | A T G C A G C T C A G T G T A C A G T C A C G T A G T C G C A T G C T A G A C T | 1e-1178 | -2713.548265 | 93.08% | 30.19% | motif file (matrix) |
| 13 | 0.886 | A T G C A C G T A C G T A G T C A G T C A G C T A G T C | 1e-987 | -2274.234935 | 77.62% | 20.25% | motif file (matrix) |
| 14 | 0.915 | C T A G T C G A T C A G C T A G C T G A G C T A T A C G A G C T C T A G T C A G | 1e-974 | -2243.785783 | 88.55% | 30.58% | motif file (matrix) |
| 15 | 0.883 | C T G A A T G C A C G T A C G T A G T C A G T C A G T C T G A C G T A C | 1e-876 | -2018.124453 | 89.98% | 35.18% | motif file (matrix) |
| 16 | 0.852 | A G T C C G T A A T G C A C G T A C G T A G T C A G T C | 1e-866 | -1994.773695 | 73.03% | 19.60% | motif file (matrix) |
| 17 | 0.870 | C T G A A C T G A T C G C G T A C G T A T A C G A C G T C T A G T A C G C G A T C G T A | 1e-792 | -1825.341215 | 49.41% | 7.34% | motif file (matrix) |
| 18 | 0.855 | A T G C A C G T A C G T A G T C A T G C A C G T A G T C G C A T | 1e-781 | -1800.528139 | 60.31% | 13.08% | motif file (matrix) |
| 19 | 0.873 | A G T C G T A C C G T A A C T G A C G T A C G T G T A C A G T C A G C T A T G C C G A T G A C T A C G T | 1e-758 | -1746.570606 | 61.89% | 14.51% | motif file (matrix) |
| 20 | 0.898 | T C G A C T G A C G T A C G T A C T A G C T A G C T A G A C T G C G T A C G T A T C A G | 1e-714 | -1645.433752 | 39.05% | 4.28% | motif file (matrix) |
| 21 | 0.813 | C T G A A C G T A C G T A C G T A G T C A T G C A G C T | 1e-623 | -1436.551788 | 81.57% | 34.43% | motif file (matrix) |
| 22 | 0.867 | G C T A C G T A T A C G T C A G T C A G A C T G C T G A T G C A | 1e-581 | -1339.898335 | 70.63% | 25.66% | motif file (matrix) |
| 23 | 0.831 | C T A G C A T G A T C G T A C G C G T A G C T A T C A G A C G T | 1e-536 | -1236.455578 | 46.28% | 10.27% | motif file (matrix) |
| 24 | 0.789 | G T A C T C G A A G T C A C G T A C G T G T A C A G T C T A G C A G T C | 1e-329 | -758.407220 | 37.56% | 10.44% | motif file (matrix) |
| 25 | 0.805 | G C A T A T C G G C A T A C G T A C G T A G T C A G T C A C G T A T G C A C G T A C G T A G C T G C A T A G T C | 1e-284 | -655.758815 | 21.89% | 3.65% | motif file (matrix) |
| 26 | 0.830 | A T G C A C G T G C A T A G T C A G T C G T A C A G T C | 1e-282 | -649.508796 | 60.37% | 28.91% | motif file (matrix) |
| 27 | 0.788 | T A G C C G A T C G A T A G T C G A T C C G A T T A C G C G A T A C G T A G C T G C A T C G A T | 1e-267 | -615.800543 | 21.96% | 3.98% | motif file (matrix) |
| 28 | 0.870 | C A T G C T G A C T G A C T G A T C G A C G T A C T A G C T G A C T A G A C T G C G T A C G T A T A C G A T G C | 1e-246 | -568.062327 | 16.70% | 2.26% | motif file (matrix) |
| 29 | 0.750 | T A C G A G T C C G A T G C A T T A G C G T A C G C A T A T G C C G T A G C T A G C T A | 1e-239 | -552.464235 | 16.06% | 2.13% | motif file (matrix) |
| 30 | 0.762 | C T G A C T G A C G T A C G T A C T A G T A C G C G T A A C T G C G T A C G T A T A C G G C A T T C A G | 1e-232 | -534.798618 | 19.65% | 3.65% | motif file (matrix) |
| 31 | 0.758 | C A G T A G C T A G T C G T A C G A T C G T A C A G C T C G A T A C G T A C T G G T A C A G C T | 1e-164 | -379.380699 | 11.18% | 1.48% | motif file (matrix) |
| 32 | 0.783 | C T G A A G C T A C G T A G T C A G T C A G T C A G T C A G C T A G C T C G A T A C G T A G C T A C G T | 1e-103 | -238.087494 | 7.44% | 1.06% | motif file (matrix) |
| 33 | 0.751 | T G C A G A T C G C A T G C A T T A G C G T A C T G A C G C A T G C A T C A G T A G C T C G A T | 1e-94 | -218.444718 | 6.23% | 0.77% | motif file (matrix) |
| 34 | 0.750 | A G T C A C G T C G A T A G T C A G T C G T A C G A T C G C A T C G A T A C G T A C T G G T A C A C G T | 1e-62 | -144.209331 | 4.01% | 0.47% | motif file (matrix) |
| 35 | 0.760 | A C T G A C G T A C G T A G T C A G T C A G T C A G T C A G T C | 1e-26 | -61.420617 | 7.32% | 3.25% | motif file (matrix) |
